# Transmission of SARS-CoV-2 within households: a remote prospective cohort study in European countries

**DOI:** 10.1007/s10654-022-00870-9

**Published:** 2022-05-28

**Authors:** Janneke D. M. Verberk, Marieke L. A. de Hoog, Ilse Westerhof, Sam van Goethem, Christine Lammens, Greet Ieven, Erwin de Bruin, Dirk Eggink, Julia A. Bielicki, Samuel Coenen, Janko van Beek, Marc J. M. Bonten, Herman Goossens, Patricia C. J. L. Bruijning-Verhagen

**Affiliations:** 1 Julius Centre for Health Sciences and Primary Care, Department of Epidemiology, University Medical Centre Utrecht, PO Box 85500, 3508 GA Utrecht, The Netherlands; 2 Department of Medical Microbiology and Infection Prevention, University Medical Centre Utrecht, Utrecht, The Netherlands; 3 Laboratory of Medical Microbiology, Vaccine and Infectious Disease Institute, University of Antwerp, Antwerp, Belgium; 4 Department of Viroscience, Erasmus University Medical Center Rotterdam, Rotterdam, The Netherlands; 5 Centre for Infectious Disease Control, WHO COVID-19 Reference Laboratory, National Institute for Public Health and the Environment (RIVM), Bilthoven, The Netherlands; 6 Infection Prevention and Control, University of Basel Childrens Hospital, Basel, Switzerland

**Keywords:** COVID-19 (household) transmission, Epidemiology, SARS-CoV-2

## Abstract

Household transmission studies are useful to quantify SARS-CoV-2 transmission dynamics. We conducted a remote prospective household study to quantify transmission, and the effects of subject characteristics, household characteristics, and implemented infection control measures on transmission. Households with a laboratory-confirmed SARS-CoV-2 index case were enrolled < 48 h following test result. Follow-up included digitally daily symptom recording, regular nose-throat self-sampling and paired dried blood spots from all household members. Samples were tested for virus detection and SARS-CoV-2 antibodies. Secondary attack rates (SARs) and associated factors were estimated using logistic regression. In 276 households with 920 participants (276 index cases and 644 household members) daily symptom diaries and questionnaires were completed by 95%, and > 85% completed sample collection. 200 secondary SARS-CoV-2 infections were detected, yielding a household SAR of 45.7% (95% CI 39.7–51.7%) and per-person SAR of 32.6% (95%CI: 28.1-37.4%). 126 (63%) secondary cases were detected at enrollment. Mild (aRR = 0.57) and asymptomatic index cases (aRR = 0.29) were less likely to transmit SARS-CoV-2, compared to index cases with an acute respiratory illness (*p* = 0.03 for trend), and child index cases (< 12 years aRR = 0.60 and 12-18 years aRR = 0.85) compared to adults (*p* = 0.03 for trend). Infection control interventions in households had no significant effect on transmission. We found high SARs with the majority of transmissions occuring early after SARS-CoV-2 introduction into the household. This may explain the futile effect of implemented household measures. Age and symptom status of the index case influence secondary transmission. Remote, digitally-supported study designs with self-sampling are feasible for studying transmission under pandemic restrictions.

**Supplementary Information:**

The online version contains supplementary material available at 10.1007/s10654-022-00870-9.

## Introduction

Households remain an important setting for severe acute respiratory syndrome coronavirus 2 (SARS-CoV-2) transmission that sustain the pandemic [1, 2]. Understanding drivers of household transmission and identifying effective household interventions to reduce transmission are, therefore, important for continued epidemic control. As SARS-CoV-2 infection can present with mild symptoms or occur asymptomatic, in particular in younger individuals, SARS-CoV-2 testing irrespective of symptoms is essential to quantify household transmission and for obtaining unbiased effect estimates of factors influencing transmission.

The type and frequency of samples taken within household transmission studies varies and contributes widely to the variation of secondary attack rates (SARs) found within previous studies [3–8]. Overall, the denser the sampling, the higher the SAR. For example, two household studies from the USA and the Netherlands found a per-person SAR of 53% and 43%, respectively, presumably owing to the dense sampling of both symptomatic and asymptomatic individuals with regular real-time reverse transcription polymerase chain reaction (RT-PCR) and serology testing [3, 4]. These estimates are much higher than the reported average per-person SAR of 16.6% based on a meta-analysis by Madewell et al. [5] who included mainly studies using passive surveillance data of PCR tests only. Household transmission may also depend on socio-cultural factors and living conditions, therefore results may not be generalizable between settings or regions.

We conducted a prospective study in the Netherlands, Belgium and Switzerland between April 2020 and April 2021, investigating transmission from confirmed SARS-CoV-2 index patients to household members. For infection control purposes, this study was set up fully remote and used regular self-sampling by study participants. This omits the requirement for physical contact between the SARS-CoV-2 infected household members and study personnel. The aim of this study was to estimate SARs. Second, factors that impact transmission were determined, with a specific focus on the effect of the infection control measures taken in households in the Western European setting.

## Methods

### Study design and data collection

Within this prospective cohort study, households with at least two members were recruited by the University Medical Centre Utrecht (UMCU), the Netherlands, the University Hospital of Antwerp (UA), Belgium and the University Children’s Hospital Basel (UCHB), Switzerland either via symptomatic healthcare worker screening programs for SARS-CoV-2, drive-through or walk-in testing sites, general practitioner visits or pre-operative screening programs. Households were eligible following a first laboratory-confirmed positive SARS-CoV-2 RT-PCR test result in a household member (index case) and enrolled within 48 h following test result. The medical ethical committees of all three sites approved the study. Written informed consent was obtained from all participating household members or their legal guardians.

A courier delivered sample kits at home addresses without entering the home. Household members received login details and instructions by email to download the study App (COVapp), which is a custom-made application compatible with Apple and Android systems, developed by the UMCU in collaboration with YourResearch Holding BV. COVapp contained all study related tasks and questionnaires along with tutorial videos, FAQs and options to contact the study team. Daily App-notifications were send to participants to remind them to complete diary entry and self-sampling when applicable. Study teams received daily reports on participant non-compliance which was followed-up by email, phone or text-message.

Following enrollment, each household member was instructed to take a nose-throat swab by self-sampling at home, irrespective of symptoms, and a dried blood spot (DBS) by self-finger-prick. A stool sample on day seven was included for children aged 0–2 years. Nose-throat swabs (NTS) were repeated if household members developed symptoms of acute respiratory illness (ARI) during follow-up. Similarly, an additional stool sample was requested from children < 2 years seven days post-symptom onset. A slightly modified protocol was used at the Swiss site, without a NTS from the index case at enrollment and without collection of stool samples. Self-sampling was supported by instruction videos and leaflets delivered with the sampling material. A telephone helpdesk was available 6 days a week during working hours.

At baseline, each household member completed a questionnaire including age, comorbidities and recent respiratory complaints. A household questionnaire was completed by one adult household member, including living conditions and infection control measures taken to prevent household SARS-CoV-2 transmission. Daily follow-up included a digital diary for each participating household member detailing respiratory and systemic symptoms. Parents or caregivers completed the questionnaires and symptom diaries for their children aged < 16 years, or for family members without access to the app (Supplement Table 1). Daily follow-up was continued until 21 days after last symptom-onset in any household member. Ten days later a second DBS was collected from all household members. All data were collected by means of COVapp. All data entered in COVapp were stored in an online secured database. Data were accessible and could be navigated in real time by authorized login through and online portal by the study team. See the Supplement for more details about the data collection.

### Laboratory analyses

Nose-throat swabs and stool samples were tested for the presence of SARS-CoV-2 by RT-PCR as described elsewhere [9, 10]. Specimens with a cycle threshold (Ct) value less than or equal to 40 were defined as SARS-CoV-2 positive. DBS specimens were tested in a final dilution of 1:40 by multiplex protein microarray for IgG antibodies targeting recombinant SARS-CoV-2 spike (S) ectodomain and S1 domain subunit antigens expressed in HEK293 cells as described elsewhere [11–13]. The S1 antigen signal exceeding 13,000 relative fluorescence units (RFU) and a S ectodomain exceeding 2,000 RFU were considered positive (for details see Supplement).

### Case definitions

A new ARI episode based on daily symptom reports was defined as: (1) new onset of fever or (2) two consecutive days with at least (a) one respiratory symptom (cough, sore throat, cold, dyspnea) and one systemic symptom (headache, muscle ache, cold shivers or fatigue) or; (b) two respiratory symptoms.

A SARS-CoV-2 infected case was defined as (1) a positive RT-PCR result from nose-throat or fecal sample, or a documented positive test result from external source (i.e. municipal testing facility;) or (2) SARS-CoV-2 negative serology at enrollment and positive serology at end of follow-up (seroconversion); or (3) positive serology at enrollment and recent (< 2 weeks before enrollment) respiratory symptoms. For each case, the symptom status was classified as ARI (meeting ARI case definition); mildly symptomatic (some respiratory or systemic symptoms but not meeting ARI threshold) or asymptomatic (no symptoms reported).

A secondary case was defined as any SARS-CoV-2 infection in a household member not being the index case, detected at enrollment or during follow-up, regardless of symptom onset.

### Statistical analysis

#### Secondary attack rate

Household SAR was calculated by dividing the number of households with at least one secondary case by the total number of participating households. The per-person SAR was calculated by dividing the number of secondary cases by the number of household members at risk (i.e. excluding the index case). To account for within household clustering the per-person SAR was estimated using generalized estimating equations (GEE) with a log link function and exchangeable correlation structure. Household members with an unknown secondary case status (i.e. no test result available to determine absence or presence of SARS-CoV-2 infection) were excluded from analysis.

We explored household, household member- and index case characteristics associated with SARS-CoV-2 transmission, as well as the impact of household infection control measures on transmission using univariate and multivariable analyses. Characteristics and measures with a univariate *p*-value of < 0.1 were included in multivariable analysis. The outcomes represents a relative risk (RR).

We conducted three sensitivity-analyses; (1) Household and per-person SAR were calculated excluding household members with a positive RT-PCR at enrollment to exclude possible co-primary cases jointly infected by an external source. (2) For comparison of our results with other (retrospective) household studies that most commonly apply PCR testing for symptomatic subjects only and without serological testing, we calculated household and per-persons SAR including only symptomatic secondary cases with positive RT-PCR result. (3) The effect of implemented infection control measures was recalculated excluding households with a co-primary case as transmission had probably already occurred within these households.

All analyses were performed in R version 4.0.3 (R Foundation for Statistical Computing, Vienna, Austria).

## Results

From April 2020 until April 2021, a total of 276 households were enrolled including 920 subjects (276 index cases and 644 household members; Table 1 and 2): 208 households at UMCU, 32 at UA and 36 at UCHB. 173 (62.6%) households were enrolled during the first epidemic wave between September and November 2020. The median household size was 3 persons (IQR: 2–4) and the majority were families (68.3%). The median duration of daily follow-up per household was 36 days (IQR: 31–46 days). In total 877 (95.3%) participant diaries and > 98% of the baseline household and participant questionnaires were completed. In addition, 80–95% of the requested NTS and DBS samples were collected. Sample completeness depended on the type and timing of the requested sample. Analysis of 167 (18.2%) DBS at enrollment and 85 (9.2%) at study completion failed because they were insufficiently saturated leaving too little volume for analysis. See Supplement Table 2 and Table 3 for more details.

**Table 1:** Characteristics of 276 households and index cases

Household characteristics	No. (%)^$^					*p*-value
	Total households (*N* = 276)	Households with secondary transmission (*N* = 126)	Households without secondary transmission (*N* = 150)	Household SAR % (95% CI)	Univariable RR (95% CI)	
Household size						0.17
2 persons	84 (31.1%)	36 (29.0%)	48 (32.9%)	42.9 (32.3–54.1)	Ref	
3 persons	54 (20.0%)	19 (15.3%)	35 (24.0%)	35.2 (23.0–49.4)	0.82 (0.51–1.25)	
4 persons	77 (28.5%)	41 (33.1%)	36 (24.7%)	53.2 (41.6–64.6)	1.24 (0.90–1.73)	
≥ 5 persons	55 (20.4%)	28 (22.6%)	27 (18.5%)	50.9 (37.2–64.5)	1.19 (0.82–1.70)	
Type of household						0.41
Family	185 (68.3%)	88 (70.4%)	97 (66.4%)	47.6 (40.2–55.0)	Ref	
Couple	62 (22.9%)	29 (23.2%)	33 (22.6%)	46.8 (34.2–59.8)	0.98 (0.71–1.31)	
Student house/cohabiting friends/ Other	24 (8.9%)	8 (6.4%)	16 (11.0%)	33.3 (16.4–55.3)	0.70 (0.35–1.15)	
Educational level^¥^						0.51
High	220 (80.3%)	99 (78.6%)	121 (81.8%)	45.0 (38.3–51.8)	Ref	
Middle/low	54 (19.7%)	27 (21.4%)	27 (18.2%)	50.0 (37.1–62.9)	1.11 (0.80–1.47)	
Mean number of bedrooms per person (SD)	1.1 (0.4)	1.0 (0.4)	1.1 (0.4)		0.94 (0.66–1.28)	0.72
Number of toilets						0.77
1	59 (21.8%)	25 (20.0%)	34 (23.3%)	42.4 (29.8–55.9)	Ref	
2	172 (63.5%)	82 (65.6%)	90 (61.6%)	47.7 (40.1–55.4)	1.13 (0.82–1.62)	
> 2	40 (14.8%)	18 (14.4%)	22 (15.1%)	45.0 (29.6–61.3)	1.06 (0.66–1.67)	
Pet owner						0.34
No pets	144 (53.3%)	70 (56.5%)	74 (50.7%)	48.6 (40.3–57.0)	Ref	
Any pets	126 (46.7%)	54 (43.5%)	72 (49.3%)	42.9 (34.2–52.0)	0.88 (0.67–1.14)	
Inclusion site						0.24
UA (Belgium)	32 (11.6%)	19 (15.1%)	13 (8.7%)	59.4 (40.8–75.8)	Ref	
UCHB (Switzerland)	36 (13.0%)	15 (11.9%)	21 (14.0%)	41.7 (26–59.1)	0.70 (0.42–1.13)	
UMCU (Netherlands)	208 (75.4%)	92 (73.0%)	116 (77.3%)	44.2 (37.4–51.3)	0.74 (0.56–1.08)	
Inclusion month						0.29
Winter (Dec–Feb)	60 (21.7%)	33 (26.2%)	27 (18.0%)	55 (41.7–67.7)	Ref	
Spring (March–May)	30 (10.9%)	15 (11.9%)	15 (10.0%)	45.8 (26.2–66.8)	0.91 (0.57–1.36)	
Summer (June–August)	17 (6.2%)	6 (4.8%)	11 (7.3%)	35.3 (15.3–61.4)	0.64 (0.28–1.15)	
Autumn (Sept–Nov)	169 (61.2%)	72 (57.1%)	97 (64.7%)	42.6 (35.1–50.4)	0.77 (0.59–1.05)	
Index case characteristics						
Age group, years						0.06
> 18	229 (83.3%)	111 (88.8%)	118 (78.7%)	48.5 (41.9–55.1)	Ref	
12–18	26 (9.5%)	9 (7.2%)	17 (11.3%)	34.6 (17.9–55.6)	0.71 (0.38–1.14)	
< 12	20 (7.3%)	5 (4.0%)	15 (10.0%)	25.0 (9.6–49.4)	0.52 (0.20–0.97)	
Sex						0.50
Female	176 (63.8%)	83 (65.9%)	93 (62.0%)	47.2 (39.6–54.8)	Ref	
Male	100 (36.2%)	43 (34.1%)	57 (38.0%)	43.0 (33.3–53.3)	0.91 (0.68–1.19)	
BMI*						0.56
Age < 2 years	1 (0.4%)	1 (0.8%)	0 (0.0%)			
Normal weight	151 (56.1%)	74 (59.7%)	77 (53.1%)	49 (40.8–57.2)	Ref	
Underweight	3 (1.1%)	1 (0.8%)	2 (1.4%)	33.3 (1.8–87.5)	0.68 (0.14–3.40)	
Overweight	85 (31.6%)	34 (27.4%)	51 (35.2%)	40 (29.7–51.2)	0.82 (0.60–1.11)	
Obesity	29 (10.8%)	14 (11.3%)	15 (10.3%)	48.3 (29.9–67.1)	0.99 (0.65–1.48)	
Underlying medical condition						0.56
No underlying medical condition	242 (89.3%)	114 (90.5%)	128 (88.3%)	47.1 (40.7–53.6)	Ref	
Any underlying medical condition	29 (10.7%)	12 (9.5%)	17 (11.7%)	41.4 (24.1–60.9)	0.88 (0.52–1.30)	
Symptom status						0.002
ARI	205 (75.1%)	106 (84.1%)	99 (67.3%)	51.7 (44.7–58.7)	Ref	
Mild symptoms	59 (21.6%)	19 (15.1%)	40 (27.2%)	32.2 (21–45.8)	0.62 (0.40–0.89)	
Asymptomatic	9 (3.3%)	1 (0.8%)	8 (5.4%)	11.1 (0.6–49.3)	0.21 (0.01–0.79)	
Hospitalized at time of enrollment						0.81
Yes	8 (2.9%)	4 (3.2%)	4 (2.7%)	50.0 (21.5–78.5)	Ref	
No	265 (97.1%)	121 (96.8%)	144 (97.3%)	45.7 (39.6–51.9)	0.91 (0.45–1.85)	

^$^Some numbers might not add up to 276 due to missing values

^¥^Educational level was categorized as high if at least one household member aged ≥ 21 years had completed at least vocational or university education and middle/low for all others

^*^BMI only available for index cases ≥ 2 years. BMI categories for index cases 2–20 year defined as BMI z-score < -2 = underweight, BMI z-score -2–1 = normal weight, BMI z-score 1–2 = overweight, BMI z-score > 2 = obesity. BMI categories for index cases ≥ 21 years defined as BMI < 18.5 = Underweight, BMI 18.5–25 = Normal weight, BMI 25–30 = Overweight" & BMI > 30 = Obesity [47]

Abbreviations: 95% CI: 95% confidence interval, SAR: Secondary attack rate, RR: Relative Risk, SD: standard deviation, BMI: Body Mass Index, UA: University Hospital of Antwerp, UCHB: University Children’s Hospital Basel University, UMCU: University Medical Centre Utrecht

**Table 2:** Characteristics of the household members *n* = 644

Characteristics	No. (%)^$^					*P*-value Univariable RR	Multivariable RR (95% CI)
	Total	Secondary case	No secondary case	Per person SAR (95% CI)^**¥**^	Univariable RR (95% CI)		
Household members at risk	644	200	444				
Age group in years						0.60	
> 18	394 (61.3%)	129 (64.5%)	265 (59.8%)	33.8 (28.8–39.2)	Ref		
12–18	107 (16.6%)	28 (14.0%)	79 (17.8%)	29.4 (21.1–39.3)	0.87 (0.63–1.19)		
< 12	142 (22.1%)	43 (21.5%)	99 (22.3%)	30.9 (23.3–39.7)	0.91 (0.70–1.19)		
Sex						0.30	
Female	315 (48.9%)	93 (46.5%)	222 (50.0%)	31.0 (25.7–36.8)	Ref		
Male	329 (51.1%)	107 (53.5%)	222 (50.0%)	34.0 (28.8–39.7)	1.10 (0.92–1.32)		
Underlying medical condition						0.37	
No underlying medical condition	566 (89.1%)	174 (87.0%)	392 (90.1%)	32.4 (27.8–37.4)	Ref		
Any underlying medical condition	69 (10.9%)	26 (13.0%)	43 (9.9%)	37.1 (27.0–48.4)	1.14 (0.85–1.54)		
Relationship to index case						**0.01**	
Spouse	171 (26.9%)	71 (36.6%)	100 (22.6%)	42.3 (35.2–49.7)	Ref		Ref
Child	211 (33.2%)	66 (34.0%)	145 (32.8%)	33.4 (26.3–41.3)	0.79 (0.62–1.00)		0.78 (0.62–0.98)
Parent	122 (19.2%)	28 (14.4%)	94 (21.3%)	22.5 (14.8–32.7)	0.53 (0.35–0.82)		0.66 (0.37–1.19)
Siblings	79 (12.4%)	16 (8.2%)	63 (14.3%)	19.5 (11.3–31.5)	0.46 (0.27–0.79)		0.58 (0.31–1.10)
Friend/other housemate	53 (8.3%)	13 (6.7%)	40 (9.0%)	22.6 (12.2–38.2)	0.54 (0.29–0.97)		0.50 (0.28–0.89)
Index case age group, years						**0.03**	
> 18	489 (76.6%)	172 (86.4%)	317 (72.2%)	36.2 (31.1–41.7)	Ref		Ref
12–18	85 (13.3%)	19 (9.5%)	66 (15.0%)	22.6 (12.0–38.4)	0.62 (0.34–1.14)		0.85 (0.39–1.85)
< 12	64 (10.0%)	8 (4.0%)	56 (12.8%)	13.4 (5.3–29.9)	0.37 (0.15–0.90)		0.60 (0.20–1.82)
Index case sex						0.95	
Female	400 (62.1%)	119 (59.5%)	281 (63.3%)	32.4 (27.0–38.4)	Ref		
Male	244 (37.9%)	81 (40.5%)	163 (36.7%)	33.8 (25.3–41.3)	1.01 (0.75–1.37)		
Index case BMI*						0.23	
Age < 2 years	4 (0.6%)	1 (0.5%)	3 (0.7%)	-	-		
Normal weight	367 (58.6%)	123 (62.1%)	244 (57.0%)	35.8 (29.5–42.6)	Ref		
Underweight	9 (1.4%)	1 (0.5%)	8 (1.9%)	11.1 (0.2–43.1)	0.31 (0.06–1.55)		
Overweight	192 (30.7%)	54 (27.3%)	138 (32.2%)	28.0 (20.8–36.6)	0.78 (0.56–1.10)		
Obesity	54 (8.6%)	19 (9.6%)	35 (8.2%)	38.3 (24.2–54.7)	1.07 (0.68–1.68)		
Index case having any underlying medical condition						0.57	
No	572 (90.1%)	184 (92.0%)	388 (89.2%)	33.6 (28.7–38.8)	Ref		
Yes	63 (9.9%)	16 (8.0%)	47 (10.8%)	29.0 (17.3–44.5)	0.87 (0.53–1.43)		
Index case symptom status						**0.01**	
ARI	482 (75.3%)	171 (85.5%)	311 (70.7%)	37.3 (31.9–43.0)	Ref		Ref
Mild symptoms	132 (20.6%)	27 (13.5%)	105 (23.9%)	21.3 (13.8–31.5)	0.57 (0.37–0.89)		0.57 (0.36–0.90)
Asymptomatic	26 (4.1%)	2 (1.0%)	24 (5.5%)	8.0 (1.2–38.9)	0.21 (0.03–1.35)		0.29 (0.05–1.89)
Index case hospitalized at time of enrollment						0.87	
Yes	21 (3.3%)	6 (3.0%)	15 (3.4%)	30.4 (11.1–60.5)	Ref		
No	620 (96.7%)	193 (97.0%)	427 (96.6%)	32.6 (28.0–37.6)	1.07 (0.44–2.61)		
Inclusion season						0.20	
Winter (Dec–Feb)	154 (23.9%)	63 (31.5%)	91 (20.5%)	41.1 (31.0–51.9)	Ref		
Spring (March–May)	78 (12.1%)	23 (11.5%)	55 (12.4%)	33.8 (21.5–48.7)	0.82 (0.51–1.34)		
Summer (June–August)	34 (5.3%)	7 (3.5%)	27 (6.1%)	23.9 (10.7–45.1)	0.58 (0.27–1.27)		
Autumn (Sept–Nov)	378 (58.7%)	107 (53.5%)	271 (61.0%)	29.8 (24.3–35.9)	0.73 (0.53–1.00)		
Inclusion site						0.32	
UA (Belgium)	74 (11.5%)	26 (13.0%)	48 (10.8%)	40.5 (27.8–54.5)	Ref		
UCHB (Switzerland)	103 (16.0%)	30 (15.0%)	73 (16.4%)	26.9 (16.7–40.2)	0.66 (0.38–1.16)		
UMCU (Netherlands)	467 (72.5%)	144 (72.0%)	323 (72.7%)	32.5 (27.3–38.3)	0.80 (0.55–1.17)		

^$^Some numbers might not add up to 644 due to missing values

^¥^Per-person SARs and related relative risks are estimated by using GEE models taking into account clustering within households

^*^BMI only available for index cases ≥ 2 years. BMI categories for index cases 2–20 year defined as BMI z-score < -2 = underweight, BMI z-score -2–1 = normal weight, BMI z-score 1–2 = overweight, BMI z-score > 2 = obesity. BMI categories for index cases ≥ 21 years defined as BMI < 18.5 = Underweight, BMI 18.5–25 = Normal weight, BMI 25–30 = Overweight" & BMI > 30 = Obesity [47]

Abbreviations: 95% CI: 95% confidence interval, SAR: Secondary attack rate, RR: Relative Risk, BMI: Body Mass Index, UA: University Hospital of Antwerp, UCHB: University Children’s Hospital Basel University, UMCU: University Medical Centre Utrecht

The median age of index cases was 36 years (IQR: 24–50 years), 46 (16.8%) were < 18 years (Table 1). The median time between index symptom onset and positive test result was 2 days (IQR: 1–4 days) and study enrollment started a median of 4 days (IQR: 3–5 days) after symptom onset. In total 264 index cases developed symptoms, while nine remained asymptomatic throughout the study. Of symptomatic index cases, six (2.3%) were pre-symptomatic at enrollment. For 14 (5.1%) index cases the information on symptoms was missing at enrollment.

**Fig. 1:**
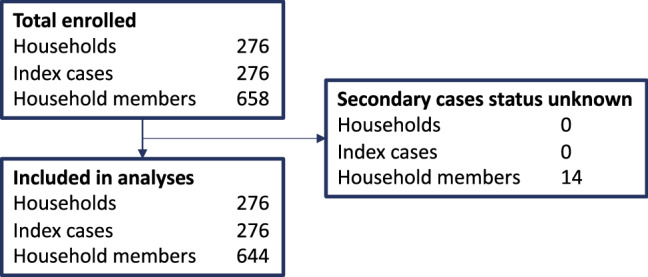
Flow diagram of the study population

Out of 658 household members, 14 were excluded from further analyses because their secondary case status could not be determined (Fig. 1). Among the 498 household members with serology at baseline, 24 (4.8%) had evidence of previous infection (detectable SARS-CoV-2 IgG antibodies at enrollment and no respiratory symptoms in the past 2 weeks). Of these, two (8.3%) also had a positive RT-PCR at enrollment (CT-values 21.4 and 32.1), possibly indicating asymptomatic re-infection.

**Fig. 2:**
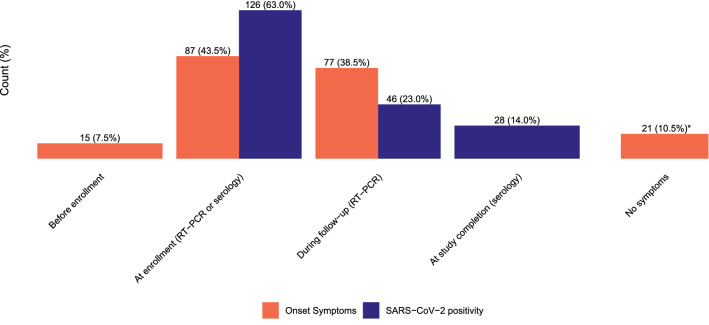
Timing of SARS-CoV-2 positivity (in blue) and symptom onset (in orange) of the secondary cases over time (*n* = 200). *Of the 21 secondary cases with no symptoms 11 (52.4%) were detected by a positive PCR at enrollment and 10 (47.6%) by seroconversion only

Of the 644 household members at risk, 200 classified as secondary case. Of these, 166 (25,8%) were detected based on positive PCR, 28 (4.4%) by seroconversion only and 6 (0.9%) by positive serology at enrollment in combination with recent (< 2 weeks ago) respiratory complaints. In total 126 (63.0%) secondary cases had a SARS-CoV-2 positive PCR test at enrollment (i.e. possible co-primary cases, Fig. 2). Respiratory symptoms at enrollment were present in 87 (43.5%) of them and 15 (7.5%) reported that symptoms had already started before enrollment. Of the 46 (23.0%) secondary cases detected post-enrollment, 33 (71.7%) took place in the first 5 days of follow-up, 10 (21.7%) between day 6–10 and 3 (6.5%) after day 10. In total 28 (14.0%) infections were detected by seroconversion only (Table 2).

### Secondary attack rates

Household secondary transmission was detected in 126 out of 276 households resulting in a household SAR of 45.7% (95% CI 39.7–51.7%; Table 3). In total, 200 secondary cases were detected among 644 household members (per-person SAR: 32.6%; 95% CI 28.1–37.4%; Table 3). Child index cases were less likely to transmit SAR-CoV-2 (per-person SAR: 13.4%; 95% CI 5.3–29.9% for children aged < 12 and 22.6%; 95% CI 12.0–38.4% for 12–18-year-olds) compared to adult index cases (36.2%; 95% CI 31.1–41.7%; *p* = 0.03, Table 2). The SAR was higher when the index had ARI (37.3%; 95% CI 31.9–43.0%) compared to mild symptoms (21.3%; 95% CI 13.8–31.5%) or completely asymptomatic index cases (8.0%; 95% CI 1.2–38.9%; *p* = 0.01). The SAR was lowest between children (19.5%; 95% CI 11.3–31.5%) and highest between spouses (42.3%; 95% CI 35.2–49.7%; *p* = 0.006).

**Table 3:** Estimates of transmission and proportion of asymptomatic secondary cases

Type of secondary attack rate and proportion of asymptomatic secondary cases	% (95% CI)	*n*/*N*
Household SAR†	45.7 (39.7–51.7)	126/276
Household SAR symptomatic secondary cases with positive RT-PCR^	35.9 (30.3–41.9)	99/276
Per-person SAR‡	32.6 (28.1–37.4)	200/644
Per-person SAR symptomatic secondary cases with positive RT-PCR^	19.3 (15.7–23.5)	118/644
Proportion asymptomatic secondary cases§	10.5 (6.8–15.8)	21/200

^†^Number of households with at least one secondary case divided by the number of households at risk

^Including only symptomatic and RT-PCR positive secondary cases

^‡^Per-person SAR is estimated by using a GEE model taking into account clustering in households

^§^Number asymptomatic divided by the total number of secondary cases

Table 4 shows household SARs by implemented infection control measures. The SAR was lower in households where members reported use of surgical masks (30.8%; 95% CI 17.5–47.7% versus 48.7%; 95% CI 42.1–55.3% without surgical masks, *p* = 0.04). Other measures, or the cumulative number of measures implemented were not significantly associated with household transmission.

**Table 4:** Impact of preventive measures in households

Prevention measures	Total number of households (*n* = 276)	Households with secondary transmission (*n* = 126)	Households without secondary transmission (*n* = 150)	Secondary attack rate (95% CI)	Univariable RR (95% CI)	*P*-value univariable RR	Multivariable RR (95% CI)
Number of implemented measures (mean (SD))	4.9 (2.6)	4.7 (2.5)	5.0 (2.7)		0.98 (0.93–1.03)	0.34	
Sleeping solely (*n*, (%))						0.91	
No	92 (33.9%)	42 (33.6%)	50 (34.2%)	45.7 (35.3–56.3)	Ref		
Yes	179 (66.1%)	83 (66.4%)	96 (65.8%)	46.4 (38.9–53.9)	1.02 (0.78–1.35)		
Separate towels (*n*, (%))						0.80	
No	91 (33.6%)	41 (32.8%)	50 (34.2%)	45.1 (34.7–55.8)	Ref		
Yes	180 (66.4%)	84 (67.2%)	96 (65.8%)	46.7 (39.3–54.2)	1.04 (0.79–1.38)		
Extra cleaning (*n*, (%))						**0.09**	
No	96 (35.4%)	51 (40.8%)	45 (30.8%)	53.1 (42.7–63.3)	Ref		Ref
Yes	175 (64.6%)	74 (59.2%)	101 (69.2%)	42.3 (34.9–50.0)	0.80 (0.62–1.03)		0.84 (0.65–1.10)
Extra ventilation (*n*, (%))						0.98	
No	93 (34.3%)	43 (34.4%)	50 (34.2%)	46.2 (35.9–56.8)	Ref		
Yes	178 (65.7%)	82 (65.6%)	96 (65.8%)	46.1 (38.6–53.7)	1.00 (0.77–1.32)		
Separate cutlery, cups and glasses (*n*, (%))						0.31	
No	89 (32.8%)	45 (36.0%)	44 (30.1%)	50.6 (39.8–61.2)	Ref		
Yes	182 (67.2%)	80 (64.0%)	102 (69.9%)	44.0 (36.7–51.5)	0.87 (0.67–1.14)		
Surgical Mask use (*n*, (%))						**0.04**	
No	232 (85.6%)	113 (90.4%)	119 (81.5%)	48.7 (42.1–55.3)	Ref		Ref
Yes	39 (14.4%)	12 (9.6%)	27 (18.5%)	30.8 (17.5–47.7)	0.63 (0.36–0.97)		0.67 (0.38–1.04)
Private toilet (*n*, (%))						0.79	
No	152 (56.1%)	69 (55.2%)	83 (56.8%)	45.4 (37.4–53.7)	Ref		
Yes	119 (43.9%)	56 (44.8%)	63 (43.2%)	47.1 (37.9–56.4)	1.04 (0.80–1.34)		
Separate devices (computer, Ipad, phone etc.; *n*, (%))						0.18	
No	129 (47.6%)	65 (52.0%)	64 (43.8%)	50.4 (41.5–59.3)	Ref		
Yes	142 (52.4%)	60 (48.0%)	82 (56.2%)	42.3 (34.1–50.8)	0.84 (0.65–1.09)		
Separate meals (*n*, (%))						0.77	
No	147 (54.2%)	69 (55.2%)	78 (53.4%)	46.9 (38.7–55.3)	Ref		
Yes	124 (45.8%)	56 (44.8%)	68 (46.6%)	45.2 (36.3–54.3)	0.96 (0.74–1.25)		
Not cuddle/kiss (*n*, (%))						0.45	
No	62 (22.9%)	26 (20.8%)	36 (24.7%)	41.9 (29.7–55.1)	Ref		
Yes	209 (77.1%)	99 (79.2%)	110 (75.3%)	47.4 (40.5–54.4)	1.13 (0.84–1.61)		
Other (*n*, (%))						0.56	
No	254 (93.7%)	116 (92.8%)	138 (94.5%)	45.7 (39.5–52.0)	Ref		
Yes	17 (6.3%)	9 (7.2%)	8 (5.5%)	52.9 (28.5–76.1)	1.16 (0.65–1.70)		

Abbreviations: 95% CI: 95% confidence interval, RR: Relative Risk, SD: standard deviation

In multivariable analysis, the age and symptom status of the index case were significantly associated with secondary transmission. The RR for index cases aged < 12 and 12–18 was 0.60 (95%CI: (0.20-1.82) and 0.85 (95%CI: 0.39-1.85) respectively, compared to index cases aged > 18 (*p* = 0.04 for trend). The RRs for mildly and asymptomatic index cases were 0.57 (95% CI 0.36–0.90) and 0.29 (95% CI 0.05–1.89), respectively, compared to index cases with ARI symptoms (*p* = 0.03 for trend). None of the household level characteristics investigated were significantly associated with household secondary transmission. Type of infection control measures implemented were also not statistically significant in multivariate analysis.

### Sensitivity analyses

The household SAR when excluding possible co-primary cases was 32.1% (95% CI 25.6–39.4%) and the per-person SAR was 16.0% (95% CI 12.5–20.3%). Using a more restrictive testing policy of RT-PCR testing for symptomatic cases only, yielded a household SAR of 35.9% (95% CI 30.3–41.9%) and a per-person SAR of 19.3% (95% CI 15.7–23.5%).

The effect of infection control measures on secondary transmission did not change when excluding the households with co-primary cases. In the univariable analysis, use of surgical masks was associated with lower SAR (RR: 0.32; 95% CI 0.05–0.96), but this effect was no longer significant in multivariate analysis.

## Discussion

In this international prospective study of 276 index subjects with SARS-CoV-2 and 644 household members, SARS-CoV-2 transmission was demonstrated in 126 (45.7%) households and on average 32.6% of household members got infected. Transmission rates declined with age of the index case and increased with symptom severity of ARI, confirming earlier observations [5, 14–16]. Two-thirds of the secondary infections among household members had already occurred at enrollment. Preventive measures implemented in the household had no discernable protective effect against transmission. The fully remote, digitally supported study with self-sampling appeared feasible for studying transmission under pandemic restrictions.

The household and per-person SARs found in this study are at the high end of the 4% to 53% reported in previous studies [3–8, 17–22]. While this may reflect true differences in local transmission dynamics across studies, two major factors determining SAR estimates are:1) the intensity of sampling protocols used and 2) in- or exclusion of co-primary cases as a secondary case in the SAR calculation. In a review by Fung et al. [17] SAR estimates more than doubled in studies with a RT-PCR test frequency of > 2 tests compared to one test. Our study had a dense sampling and intensive follow-up, combining RT-PCR screening of all symptomatic and asymptomatic household members, repeated RT-PCR testing for new onset ARI and paired antibody testing of all subjects. This allowed detection of asymptomatic infections, and those with negative RT-PCR results. Indeed, studies that used equally or more dense sampling protocols report SARs similar to our estimates [3, 4, 6]. To quantify the effect of sampling protocols on the estimated SAR, we calculated the household and per-person SARs in our study excluding asymptomatic and RT-PCR negative subjects. This would reduce the detected SARs by 9.8% and 13.3% respectively, which is more in line with estimates from (retrospective) household studies that are based on contact tracing investigations with symptom-based RT-PCR testing alone. We therefore conclude that the use of this methodology underestimates the SAR by at least 10%. In addition, in our main analysis we considered the index case as the primary case in the household, but it is possible that other household members were infected concurrently by an external source. Considering these as co-primary cases would decline SAR by approximately 15%. This could be interpreted as the lower bound of the household and per-person SAR. Of note, all households were enrolled during a period where the more transmissible mutant strains, in particular the Delta variant and later Omicron, did not yet circulate much in the Netherlands, Belgium or Switzerland [23–26]. It is likely that household SARs will be higher for the new and more transmissible variants of SARS-CoV-2. On the other hand, households were included before the SARS-CoV-2 vaccination program was (fully) rolled out and only a small proportion of the population had prior immunity.

In line with earlier findings, household contacts of index cases with more severe respiratory symptoms are at higher risk for secondary infection [5, 7, 17, 27] and transmission was highest when the index case was above the age of 18. Our study also suggests lower transmission from child index cases compared to adults, but it must be considered that children are more often mildly or asymptomatic when infected. Undetected introduction of SARS-CoV-2 infection by a pediatric primary case will therefore be more common compared to an adult primary case, which could have led to some misclassification of the index.

The high number of co-primary cases in this study and the fact that nearly all other secondary cases turned PCR positive within the first 5 days of follow-up, indicates that household transmission predominantly takes place before or around the time of detection of the index case. It is estimated that up to 44% of transmission occurs during the pre-symptomatic period in settings with substantial household clustering [28]. This may explain why household infection control measures implemented following diagnosis were not effective in preventing transmission in our study, except for possibly some effect of masking. The use of rapid antigen self-tests at symptom onset or asymptomatic screening followed by immediate implementation of infection control measures could possibly help reduce household transmission. In a qualitative sub-study in this cohort, household members reported difficulties in maintaining measures over time [29]. Compliance may increase if the recommended period for infection control measures in the household is shortened [29] and the number of measures reduced to those with evidence of effectiveness such as wearing a facemask [30–33]. Our data do not suggest any additive effect of multiple infection control measures. Of note, isolation outside the home was not deployed as policy in the Netherlands, Belgium or Switzerland and therefore not evaluated in this study.

For infection prevention purposes, this study was set up as a fully remote, digitally supported, study. Physical contact with participants was omitted by using self-sampling methods instead of sampling by healthcare professionals as done in most SARS-CoV-2 transmission studies [3, 34, 35]. The design of this study appeared feasible with > 95% completeness of diaries and questionnaires, and 80–95% completeness of samples requested during study participation. This design is particularly suitable when studying transmission under pandemic restrictions. In addition, running a fully remote study may also be more feasible in remote areas or when study participants cover a large geographical area and repeated study visits are too expensive or logistically unfeasible.

Our study has some limitations that merit discussion. First, many household members already tested positive at enrollment. As discussed above, it is possible that other household members were infected concurrently by an external source (co-primary case), overestimating the SAR. Furthermore, it is possible that some transmission between household members occurred in the opposite direction. In particular, asymptomatic or mild infections could have remained undetected until a second fully symptomatic infection occurred in the household, misclassifying primary and secondary cases. This in turn, can bias estimates on index or household contact characteristics influencing SAR. In particular the relative infectiousness of children versus adult index cases is difficult to assess without complete (asymptomatic) index case ascertainment. Alternative enrollment criteria, for instance based on household exposure rather than confirmed infection, would be needed to improve differentiation between primary and secondary cases and reconstruction of transmission chains. Second, we assumed that household transmission was responsible for all infections among household contacts. The household SAR could therefore be overestimated. However, with quarantine and isolation orders in place, community exposure among household contacts was probably minimal. Third, despite our extensive sampling protocol, it is likely that some infections were missed, thereby underestimating the SAR. In particular, asymptomatic or subjects with mild symptoms may have a weak or undetectable humoral response [36, 37] and such infections may have been missed if they occurred post-enrollment. In addition, we cannot exclude some loss in sensitivity due to self-collecting of NTS, although earlier studies suggest minimal impact on detection as compared to samples collected by healthcare professionals [38]. Similarly, self-collected DBS samples may be less sensitive than serum samples, although they have been proven a valid alternative for serum [39–41]. Serology analysis of 252 (13.7%) DBS samples failed because of to little blood volume. Choosing alternative ways of collecting blood samples, e.g. using tubes instead of DBS or samples taken by a research nurse, might improve completeness of serology results. Fourth, the study population might not be completely representative. The percentage of households with a high educational level and the percentage of healthcare workers in the study, of which the latter are dominantly women, were overrepresented compared to the average Dutch, Belgian and Swiss population [42]. Finally, this study was conducted before SARS-CoV-2 vaccination was implemented. Naturally, household transmission will be strongly influenced by presence of vaccine immunity both among index cases and among household members. [43–46].

## Conclusion

Using a fully remote, digitally supported study design we were able to study household transmission in a pandemic setting. Our study shows a high household SAR (45.7%) and per-person SAR (27.8%) for SARS-CoV-2 in the Western European context in the era before widespread circulation of alpha and delta variants and vaccine implementation. Transmission rates were associated with symptom severity and age of the index case. Most household transmission occured before or around the time of detection of the index case, with no impact of household infection control measures on transmission, except possibly for wearing a face mask.

## Supplementary Information

Below is the link to the electronic supplementary material.

Supplementary file1 (DOCX 34 KB)
