# Supplementary material for: Transmission of SARS-CoV-2 within households: a remote prospective cohort study in European countries
Source: Eur J Epidemiol. 2022 May 28;37(5):549–61. doi: 10.1007/s10654-022-00870-9 (PMC9146817; doi:10.1007/s10654-022-00870-9)
Supplement: Supplementary file 1 — Supplementary file1 (DOCX 34 KB) [file 10654_2022_870_MOESM1_ESM.docx]

**Data collection**

At study enrollment, each participant of ≥16 years received instructions to install the COVapp on a mobile device. The COVapp is a custom made application compatible with Apple and Android systems, developed by the UMCU. A unique username and password providing access to the application were sent by email. Via this application, participants could fill in several questionnaires and daily symptom diaries (self-reported; fever, coughing, sore throat, cold, dyspnea, headache, muscle pain, cold shivers, fatigue, anosmia (since July 7, 2020) and diarrhea). Parents or caregivers completed the questionnaires and symptom diaries for their children aged <16 years, or for family members without access to the app. At start of the study, per household a questionnaire was filled out containing questions regarding household characteristics and living situation. A nose-throat swab (NTS), a capillary blood sample on filter paper (dried blood spot) and a baseline questionnaire capturing demographics, comorbidities and views on prevention measures were collected from each household member irrespective of symptoms. For children aged 0-2 year, also a stool sample was collected seven days after enrollment. A daily follow-up of disease symptoms started for at least 21 days. Symptom follow-up was prolonged in case of new acute respiratory illness (ARI) episode in the household emerged. At the beginning of a new ARI episode the app generated a request for a nose-throat swab from the person with ARI disease (and an additional stool sample for children 0-2 years seven days after symptom onset) and scoring lists. At the end of the ARI episode a questionnaire about disease progression and self-reported transmission prevention behaviors popped up. Follow-up ended after 21 days or 21 days after the last date of onset of symptoms in the household. Ten days later a capillary blood sample was collected for each household member again. The mobile application automatically generated requests for swabs, blood or stools during the study. The materials for self-sampling were delivered at the beginning of the study at the home address, stored in home freezers (-15 °C) during follow-up and picked-up at the end of the study by the study team. All data entered to the app were stored in an online secured web system.

**Laboratory analysis**

At arrival on the lab blood samples and swabs were stored at -80 °C instantly until further analysis. Swabs were tested separately for the presence of SARS-CoV-2 by real-time PCR as described in detail elsewhere.[1] Briefly, 100 μl of a clinical specimen was mixed with proteinase K and 10 µl of a known amount of phocine herpesvirus (DNA) and Phocine distemper virus (RNA)—to monitor the efficient extraction of nucleic acids (NA) — and incubated for 15 min at 56°C. Subsequently, NA extraction was performed by using the specific A protocol on the NucliSens EasyMag (BioMérieux, Marcy-l’Etoile, France). The nucleic acid was then eluted in 70 μl of elution buffer and directly used for cDNA synthesis and real-time TaqMan PCR. Stool samples were mechanically pretreated by beating them through a membrane prior to the proteinase K pretreatment to optimize nucleic acid extraction. A 20-μl reaction contained 5 μl of NA extract and 5 µl of the TaqMan Fast Virus 1-Step Master Mix 4x (Thermo Fisher, Brussels, Belgium). Primer and probe sequences, as well as optimized concentrations were used as described by Corman et al.[1] All oligonucleotides were synthesized and provided by Tib Molbiol, Berlin, Germany. Thermal cycling was performed at 55°C for 10 min for reverse transcription, followed by 95°C for 3 min and then 45 cycles of 95°C for 15s, 58°C for 30s on a LightCycler 480 instrument (Roche) for the detection of the SARS-CoV-2 E-gene. In addition, NA extracts were analysed for the presence of RNAseP to check the quality of the specimens. Cycle threshold values (Ct-values) >35 were designated as invalid. The 20-µl reaction contained also 5 µl of NA extract and 5 µl of the TaqMan Fast Virus 1-Step Master Mix 4x (Thermo Fisher). Primer and probe sequences, as well as optimized concentrations were used as described by the CDC.[2] Thermal cycling was performed at 50°c for 15 min for reverse transcription, followed by 2 min at 95°C and then 45 cycles of 95°C for 15 sec and 30 sec at 55°C on the LightCycler 480 instrument (Roche). Ct-values for SARS-CoV-2 ≤40 is used as cut-off to interpreted positively.

Dried blood spot specimens were tested by multiplex protein microarray for antibodies targeting recombinant SARS-CoV-2 spike (S) ectodomain and S1 subunit antigens expressed in HEK293 cells as described earlier.[3-5] Briefly, antigens were printed on nitrocellulose coated slides and incubated for 1 hour at 37 °C with Blotto Blocker (Pierce, United States of America). Slides were washed with phosphate buffered saline (PBS) containing 0.1% Tween-20 between each step. DBS were eluted in PBS with 5% Tween-20 for one hour, diluted in Blotto Blocker with 0.1% Tween-20, and tested on the printed slides in a final 1:40 dilution for 1 hour at 37 degrees Celsius. This was followed by an incubation step with goat anti-human IgG (Fab specific) conjugated with AF647 (Jackson ImmunoResearch, United Kingdom). Slides were scanned using a Powerscanner (Tecan, Switzerland) and analyzed using ScanArray Express software (PerkinElmer, Waltham, USA). The use of DBS was compared to serum on the protein microarray platform by testing DBS specimens 1:40 and serial diluted serum specimens obtained from 8 individuals against a panel of endemic and epidemic human coronavirus recombinant antigens. The relative fluorescence units (RFU) of DBS specimens showed a good correlation and a similar level compared to 1:80 serum for various antigens. Specimens with a S1 antigen signal exceeding 13,000 relative RFU and a S ectodomain signal exceeding 2,000 RFU were considered positive.

**Supplement Table 1:** Diary items by item category.

| **Symptom category** | **Diary items** |
| --- | --- |
| Fever | Fever |
| Respiratory symptoms | Coughing |
|  | Sore throat |
|  | Cold |
|  | Dyspnea |
| Systemic symptoms | Headache |
|  | Muscle ache |
|  | Cold shivers |
|  | Fatigue |
| Other symptoms | Loss of smell/taste |
|  | diarrhea |

**Supplement Table 2:** Diary and baseline questionnaire completeness based on the 276 households (276 index cases and 644 household members).

|  | **Diaries (n=920)** | **Household baseline questionnaire (including measures) (n=276)** | **Participant baseline questionnaire (n=920)** |
| --- | --- | --- | --- |
| 100% completed | 877 (95.3%) | 271 (98.2%) | 904 (98.3%) |
| 80-100% completed | 27 (2.9%) | - |  |
| <80% completed | 10 (1.1%) | - |  |
| Not completed | 6 (0.7%) | 5 (1.8%) | 16 (1.7%) |

**Supplement Table 3:** Sampling scheme and sample completeness based on the 276 households (276 index cases and 644 household members).

|  | **Enrollment** | | | **ARI symptoms** | | **End of study** |
| --- | --- | --- | --- | --- | --- | --- |
|  | DBS | NTS† | Stool* | NTS | Stool* | DBS |
| Total samples requested | 920 | 884 | 27 | 165 | 11 | 920 |
| Missing result due to incomplete sampling n(%) | 35 (3.8%) | 48 (4.8%) | 4 (14.8%) | 21 (12.7%) | 4 (36.4%) | 58 (6.3%) |
| Missing result due to insufficient sampling n(%) | 167 (18.2%) | - | - | - | - | 85 (9.2%) |
| Not requested and collected in Basel | - | 36 | 7 | - | 1 | - |

† No PCR samples at enrollment collected for index cases in Switzerland.

*Stool samples are requested only for children aged 0-2 years 7 days after enrollment or ARI symptom onset. No stool samples collected in Switzerland.

**REFERENCES**

1. Corman VM, Landt O, Kaiser M, Molenkamp R, Meijer A, Chu DK, et al. Detection of 2019 novel coronavirus (2019-nCoV) by real-time RT-PCR. *Eurosurveillance*. 2020;25(3):2000045.

2. Real-Time RT-PCR Panel for Detection 2019-Novel Coronavirus: Centers for Disease Control and Prevention. <https://www.who.int/docs/default-source/coronaviruse/uscdcrt-pcr-panel-for-detection-instructions.pdf>. Accessed 26 March 2021.

3. de Bruin E, Loeber JG, Meijer A, Castillo GM, Cepeda ML, Torres-Sepúlveda MR, et al. Evolution of an influenza pandemic in 13 countries from 5 continents monitored by protein microarray from neonatal screening bloodspots. *J Clin Virol*. 2014;61(1):74-80.

4. Westerhuis BM, de Bruin E, Chandler FD, Ramakers CRB, Okba NMA, Li W, et al. Homologous and heterologous antibodies to coronavirus 229E, NL63, OC43, HKU1, SARS, MERS and SARS-CoV-2 antigens in an age stratified cross-sectional serosurvey in a large tertiary hospital in The Netherlands. *medRxiv*. 2020:2020.08.21.20177857.

5. Reusken CB, Haagmans BL, Müller MA, Gutierrez C, Godeke GJ, Meyer B, et al. Middle East respiratory syndrome coronavirus neutralising serum antibodies in dromedary camels: a comparative serological study. *Lancet Infect Dis*. 2013;13(10):859-66.
